# Prevalence of Hypogonadism Symptoms Among Males With Hypothyroidism at a Tertiary Hospital: A Cross-Sectional Study

**DOI:** 10.7759/cureus.50255

**Published:** 2023-12-10

**Authors:** Abdullah Alkhayal, Moeber Mahzari, Abdullah S Alhammadi, Rashad R Almutairi, Saeed M Bafaqih, Zead M Alsultan, Salman S Alqarni, Ahmed R Alibrahim

**Affiliations:** 1 Urology, King Abdulaziz Medical City, Riyadh, SAU; 2 Medicine, Ministry of the National Guard, Riyadh, SAU; 3 Medicine, King Saud Bin Abdulaziz University for Health Sciences, Riyadh, SAU; 4 Endocrinology and Metabolism, King Abdulaziz Medical City, Riyadh, SAU

**Keywords:** androgen deficiency, primary hypothyroidism, bmi, smoking, adam questionnaire and hypogonadism, high tsh and hypogonadism, adam questionnaire, smoking and hypogonadism symptoms, obesity and hypogonadism symptoms, hypogonadism and hypothyroidism

## Abstract

Background

Hypogonadism is a condition in which the body’s ability to produce sex hormones is reduced. Androgen deficiency and hypothyroidism are similar in many symptoms, and the coexistence of the two conditions is common. The aim of this study is to explore the prevalence of hypogonadism symptoms in male patients diagnosed with primary hypothyroidism.

Methods

This cross-sectional study was conducted at  King Abdulaziz Medical City  in Riyadh,  Saudi Arabia, with a sample size of 120 adult male patients with primary hypothyroidism. Data collection primarily relied on one instrument, namely, the Androgen Deficiency in Aging Males (ADAM) questionnaire, which was translated into Arabic and validated by previous researchers.

Results

A total of 120 adult males with hypothyroidism completed the ADAM questionnaire. Out of the 120 patients, 67.5% had a positive screen for hypogonadism. Among patients who had hypogonadism symptoms on the questionnaire, 81% had a BMI above 25, 69% were older than 40, and 65% were smokers.

Conclusion

Hypogonadism symptoms are common in male patients with primary hypothyroidism. Among patients with primary hypothyroidism, increasing age and being overweight added to the likelihood of having hypogonadism symptoms.

## Introduction

Androgen deficiency in aging males is a condition characterized by a decrease in the secretion of testosterone and the clinical manifestation of hypogonadism [1]. The clinical manifestations of hypogonadism share a common clinical presentation with hypothyroidism such as fatigue, increase in body fat, and muscle weakness. A common method of treatment includes hormone replacement therapy for both conditions [1-3].

Hypothyroidism and hypogonadism are well-understood individually in terms of their pathophysiology, treatment, and global epidemiology. For instance, a study conducted in Boston, Massachusetts, revealed a 5.6% prevalence of symptomatic hypogonadism, which increases with age [4]. Additionally, a meta-analysis found a mean prevalence of 3.82% for total thyroid dysfunction in Europe [5].

Research to characterize the association between primary hypothyroidism and hypogonadism is limited. However, it seems that the two conditions are associated with each other [6-8]. A study that was conducted on six male patients, all of whom presented with severe myxedema due to primary hypothyroidism, showed histological abnormalities within the testes, as well as decreased secretion of the anterior pituitary hormone gonadotropin [7]. Kumar et al. showed in a study that subclinical hypothyroidism (SCH) in adult male patients is associated with low levels of serum testosterone and its precursor progesterone [2]. Furthermore, SCH was associated with a slightly increased level of prolactin than the euthyroid group, which could decrease the bio-available testosterone [2]. Moreover, Sarma et al. reported a similar finding of association between hypogonadism and SCH [6]. Additionally, a study conducted in 2009 concluded that there is an association between hypothyroidism, decreased fertility, and sexual functions [8].

There are no data from the Middle East region on the association between hypothyroidism and hypogonadism. Therefore, the aim of this study is to investigate the prevalence of hypogonadism symptoms using the  Androgen Deficiency  in Aging Males (ADAM) questionnaire in male patients with primary hypothyroidism.

## Materials and methods

This study is a cross-sectional study conducted at King Abdulaziz Medical City in Riyadh, which is a governmental tertiary care hospital. The study involved adult male patients with primary hypothyroidism whose diagnosis was obtained retrospectively through the BESTCare system, including lab values of TSH, free T4, and T3. Furthermore, all patients had a previously established diagnosis of primary hypothyroidism for at least one year. Patients with secondary hypothyroidism were excluded, whose diagnosis was also obtained through the BESTCare system. In addition, patients under the age of 18 with primary hypothyroidism were excluded as our study was done on an adult population. The study was conducted between March 2021 and August 2022. There were 1,000 male adult hypothyroid patients available for participation in the study; however, we included a sample size of 120 patients selected using a non-probability consecutive sampling technique as the rest of the patients did not fulfill the inclusion criteria. The study was approved by King Abdullah International Medical Research Center, study number SP20/373/R. All patients signed a consent form for participation in the study.

Demographic data, such as the age, height, weight, and smoking status of the participants, were collected through the medical record system used at King Abdulaziz Medical City called BESTCare. The main tool utilized to assess the presence of hypogonadism symptoms in this study was the ADAM questionnaire. The ADAM questionnaire consists of a set of questions that identify some of the key symptoms and features of hypogonadism. A few examples of what the questionnaire included are questions on lethargy, entertainment in life, strength, weight loss, sleeping after dinner, deterioration in work performance or playing sports, and being sad or grumpy. There was an emphasis on two particular questions (1 and 7), which included an inquiry about libido and having a weaker erection. All patients filled out the ADAM questionnaire in Arabic, which was validated by Rabah et al. [9]. The questionnaire included 10 questions with yes and no answers. Each patient was considered to have significant hypogonadism symptoms if any three questions were answered with “yes” or if either question 1 or 7 were answered with "yes." The questionnaire was originally validated by Morley et al. in two studies involving aging white men [10,11]. In the first study, the St. Louis University ADAM questionnaire demonstrated a sensitivity and specificity for detecting androgen deficiency of 88% and 60%, respectively [10]. The second study had a similar sensitivity but with a lower specificity of 30% [11].

Statistical analysis

Statistical analysis was done in the Statistical Product and Service Solutions (SPSS) program version 20 (IBM SPSS Statistics for Windows, Armonk, NY). All numerical data, such as age, weight, height, and BMI, were presented as mean and standard deviation. As for the categorical variables, they were presented as frequencies and percentages. The prevalence of hypogonadism symptoms as compared between the demographic variables was done using the chi-square test. The level of significance was set at p<0.05.

## Results

There was a total of 120 adult males with primary hypothyroidism who completed the ADAM questionnaire. Table 1 shows the average levothyroxine dose for these patients (omitting seven who were off treatment) and their most recent TSH levels, as well as other values.

**Table 1:** Levothyroxine dose and recent TSH

Clinical parameters of the subjects given as different values		
	L-Thyroxine (mcg)	TSH (IU/L)
Mean	138.4	4.04
Median	125	1.76
Standard deviation	48.2	6.77
25^th^ percentile (Q1)	125	0.29
75^th^ percentile (Q3)	150	4.30

Table 2 describes the demographic characteristics of the patients. 59 (49%) were between 40 and 59 years old, and 97 (81%) had a BMI above 25. There were 83 respondents to smoking status, out of which 60 (72%) were nonsmokers.

**Table 2:** Demographic characteristics

		n	%
Smoking status	Non-smoker	60	72%
	Smoker	23	28%
Age Groups	20-39 years	37	31%
	40-59 years	59	49%
	60+ years	24	20%
BMI Group	Normal weight (<25)	23	19%
	Overweight (25-<30)	35	29%
	Obese Class I (30-<35)	40	33%
	Obese Class II/III (35+)	22	18%

Table 3 shows the prevalence of hypogonadism symptoms in hypothyroid patients based on the ADAM questionnaire, which was 81 (67.5%). In Table 3, there is also a comparison between all groups to show which variable had the most effect on developing hypogonadism symptoms. The first variable was smoking status, having 83 respondents. Out of 23 smokers, 15 (65%) had positive ADAM results. Similarly, out of the 60 non-smokers, 42 (70%) had positive ADAM results, which shows that there is no significant effect of smoking on developing hypogonadism. Three age groups were included, as described in Tables 2-3. Firstly, there were 37 patients aged from 20 to 39, out of which 24 (65%) had positive ADAM results. The second group involved 59 patients aged between 40 and 59, out of which 37 (63%) had positive ADAM results. As for the last group, 24 patients aged 60 and above, out of which 20 (83%) had positive ADAM results. This shows no statistically significant relationship between the age and developing hypogonadism, although the prevalence increased in the oldest group. On the other hand, there was a clear relation with regard to BMI. In the first group with those having a BMI that is less than 25, there were 23 patients, 13 (57%) of whom had positive ADAM results. The second group included those with a BMI between 25 and 30, and out of the 35 patients, 30 (86%) had positive ADAM results, which shows a strong relationship between being in the overweight category and developing hypogonadism symptoms. However, those who had BMI values between 30 and 35, and BMI values of 35 and above had comparable positive ADAM results (58% and 68%, respectively) to the normal BMI group (57%).

**Table 3:** Comparison between different variables in relation to hypogonadism symptoms

		Prevalence of hypogonadism symptoms				
		No hypogonadism symptoms (n=39, 32.5%)		Hypogonadism symptoms (n=81, 67.5%)		
		n	%	n	%	p-value*
Smoking status	Non-smoker	18	30%	42	70%	0.67
	Smoker	8	35%	15	65%	
Age groups	20-39 years	13	35%	24	65%	0.18
	40-59 years	22	37%	37	63%	
	60+ years	4	17%	20	83%	
BMI group	Normal weight (<25)	10	43%	13	57%	0.04
	Overweight (25-<30)	5	14%	30	86%	
	Obese Class I (30-<35)	17	43%	23	58%	
	Obese Class II/III (35+)	7	32%	15	68%	
* Chi square						

## Discussion

After utilizing the ADAM questionnaire across 120 adult primary hypothyroid patients, we found that 67.5% of them had a positive screening result for hypogonadism. Two main additional factors that were present in those who had hypogonadism symptoms included being overweight and increasing age especially those who were aged 60 years or older. These results show that there is indeed a relationship between having primary hypothyroidism and decreased gonadal function and that certain contributing factors such as age and BMI may also play a role in the link between the two disorders.

Local studies on this topic are currently not available in our geographical region; however, our results are in line with the existing literature that points to the correlation between hypothyroidism and hypogonadism [6-8]. For instance, Sarma et al. identified that men with subclinical hypothyroidism had decreased levels of total testosterone and free T4 compared to controls that were euthyroid [6]. Furthermore, in a study consisting of six adult hypothyroid patients with severe myxedema, five of whom had a prepuberal onset, testicular abnormalities were found in addition to delayed maturation owing to prolonged thyroid deficiency [7]. Many of the other studies on the link between hypothyroidism and hypogonadism we reviewed, had similar findings that showed a clear correlation between the two disorders [1,2]. Meikle found that hypogonadotropic hypogonadism associated with primary hypothyroidism was reversible with thyroid hormone replacement therapy [1].

Our study demonstrated that overweight patients with primary hypothyroidism were more likely to have hypogonadism symptoms, which suggested another implicating factor for developing hypogonadism, as seen in the literature [12-15]. For example, Molina-Vega et al. found in a study that included nondiabetic men with obesity that increasing BMI and age were independent risk factors for developing hypoandrogenemia [14]. This is surprisingly contrary to our results where patients in the obese category had a lesser prevalence of hypogonadism symptoms compared to the overweight category, which is most likely a limitation of our small sample size and lack of confirmatory laboratory testing with testosterone levels. Moreover, we found that there was no statistically significant correlation between smoking and developing hypogonadism symptoms, although 65% of those with hypogonadism symptoms were smokers. That being said, one study mentioned that smoking could mask borderline hypogonadism, and as previously mentioned, this can be missed due to the lack of confirmatory testing with testosterone [16]. Even though the prevalence of hypogonadism symptoms increased with age, especially those who were older than 65, it was not statistically significant based on our analysis which again, is most likely due to the small sample size. Nevertheless, one study we reviewed suggested that the prevalence of hypogonadism in patients older than 70 years increased compared to younger patients [8]. Additionally, some studies in our review indicated that older men who followed a healthy lifestyle and avoided gaining weight had no associated decline in serum androgens [17,18].

Despite the fact that our study lacked confirmatory laboratory testing for hypogonadism, the ADAM questionnaire proved to be a useful tool with a relatively high sensitivity for detecting hypogonadism symptoms [9-11]. Furthermore, our study is considered to be the first of its kind locally, on the prevalence of hypogonadism symptoms among adult males diagnosed with primary hypothyroidism, which will hopefully help with future focus regarding this topic.

## Conclusions

In summary, our primary goal was to build on existing research on the prevalence of hypogonadism symptoms in adults with primary hypothyroidism, particularly in Saudi Arabia. We also aimed to identify potential contributing factors such as smoking, aging, and BMI. Our study found a significant link between hypogonadism symptoms and primary hypothyroidism, consistent with existing literature. While increasing age appeared to increase the risk of hypogonadism symptoms, this finding was not statistically significant. However, being overweight was found to be a significant factor. Our study had a small sample size, which was one of the limitations we faced, but we hope our contribution helps improve understanding and clinical practices for these conditions.

## Appendices

**Table 4:** ADAM questionnaire

Name:		Age:	
		No	Yes
1. Do you have a decrease in libido (sex drive)?			
2. Do you have a lack in energy?			
3. Do you have a decrease in strength and/or endurance?			
4. Have you lost height?			
5. Have you noticed a decreased ‘enjoyment of life’?			
6. Are you sad and/or grumpy?			
7. Are your erections less strong?			
8. Have you noted a recent deterioration in your ability to play sports?			
9. Are you falling asleep after dinner?			
10. Has there been a recent deterioration in your work performance?			
